# Effects of a novel *Paraburkholderia* phage IPK on the phenanthrene degradation efficiency of the PAH-degrading strain *Paraburkholderia caledonica* Bk

**DOI:** 10.1007/s10532-025-10181-x

**Published:** 2025-09-18

**Authors:** Esteban E. Nieto, Nawras Ghanem, Robertina V. Cammarata, Felipe Borim Corrêa, Bibiana M. Coppotelli, Antonis Chatzinotas

**Affiliations:** 1 Centro de Investigación y Desarrollo en Fermentaciones Industriales, CINDEFI (UNLP; CCT-La Plata, CONICET), Street 50 N°227, 1900 La Plata, Argentina; 2 Department of Applied Microbial Ecology, Helmholtz Centre for Environmental Research - UFZ, 04318 Leipzig, Germany; 3 Institute of Biology, Leipzig University, 04103 Leipzig, Germany; 4 German Centre for Integrative Biodiversity Research (iDiv) Halle-Jena-Leipzig, 04103 Leipzig, Germany

**Keywords:** Phage-host interaction, Polycyclic aromatic hydrocarbon (PAH), Microbial degradation, Lysogeny, *Paraburkholderia caledonica*

## Abstract

Phages are a major cause of bacterial mortality, affecting bacterial diversity and ecosystem functioning. However, the impact of phage-host interactions in contaminated environments and their role in pollutant biodegradation have largely been overlooked. We isolated and characterized a novel phage that infects the PAH-degrading bacterium *Paraburkholderia caledonica* Bk from a polycyclic aromatic hydrocarbon (PAH)-contaminated soil and investigated the effect of different multiplicity of infection (MOI) ratios on the degradation efficiency of phenanthrene. The phage IPK is a temperate phage with a wide pH and temperature tolerance and a burst size of 80  PFU ml⁻^1^. The phage was classified as a member of the Caudoviricetes and is related to *Pseudomonas* and *Burkholderia* phages. However, its low intergenomic similarity indicates that it is a new species. Three auxiliary metabolic genes (AMGs) related to amino acid metabolism and to bacterial growth regulation were identified in the phage genome. The highest multiplicity of infection (MOI 10) showed a rapid recovery of the host density and greater phenanthrene degradation than MOIs ranging from 0.01 to 1. This work highlights the important role of phage-host interactions in modulating the efficiency of pollutant degradation, which could be a key for improving the establishment of inoculants in bioremediation processes.

**Graphical abstract:**

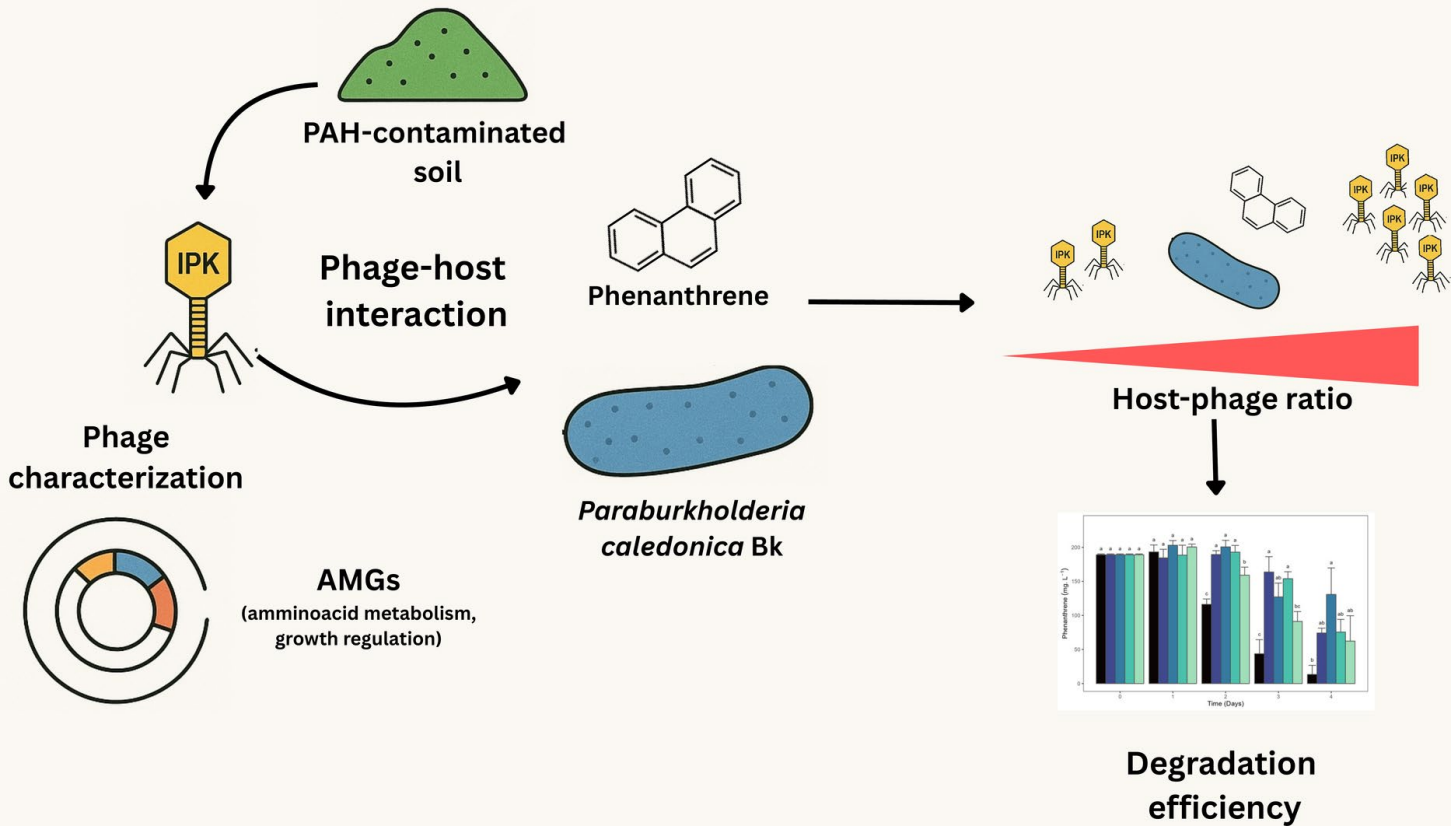

**Supplementary Information:**

The online version contains supplementary material available at 10.1007/s10532-025-10181-x.

## Introduction

Phages play a key role as a major top-down regulator of bacterial abundance, affecting bacterial diversity and ecosystem functioning. They employ a range of infection strategies, with lytic and lysogenic cycles being the best described lifestyles, though alternative strategies have also been documented (Mäntynen et al. 2021; Chevallereau et al. 2022). Lytic phages infect and kill their hosts, releasing the new progeny. Lysogenic or temperate phages can also enter into a lysogenic cycle, in which the phage genome integrates into the host chromosome as a prophage and replicates with the bacterial chromosome. Thus, while lytic phages maintain an antagonistic relationship with their hosts, the fitness of temperate phages and their hosts is more aligned, leading to more mutualistic behaviour (Obeng et al. 2016; Chevallereau et al. 2022; Jurburg et al. 2023). A common outcome of lysogeny is superinfection exclusion, where a prophage protects its host from infection by other closely related phages (Hampton et al. 2020).

Several models have been developed to describe phage-host dynamics (Knowles et al. 2016; Silveira et al. 2021; Voigt et al. 2021; Brown et al. 2022). The “Killing-the-Winner” (KtW) model proposes that lytic phages suppress fast-growing bacteria, thereby controlling bacterial community abundance and ensuring the coexistence of less competitive species (Thingstad 2000). The “Piggyback-the-Winner” (PtW) and “Piggyback-the-Loser” (PtL) models describe temperate phage dynamics and focus on the switch from the lytic to the lysogenic cycle as the host density changes. This is characterised by an increase in lysogeny at high and low bacterial densities, respectively (Knowles et al. 2016; Silveira et al. 2021). In addition, bacterial hosts indirectly control phage production through physiological factors, such as growth rate, which is linked to resource availability (Zimmerman et al. 2020).

At the ecosystem level, phages affect soil functioning and microbial diversity by causing bacterial mortality (Kuzyakov and Mason-Jones 2018) and by facilitating species coexistence (Voigt et al. 2021; Carreira et al. 2024). Phage activity also affects biogeochemical cycling through the viral shunt, as virus-induced mortality releases nutrients that are then available for uptake by other organisms (Braga et al. 2020; Carreira et al. 2024). Notably, there is some evidence that phages from polluted environments show genomic differences compared to those from unpolluted environments (Huang et al 2024). Lysogeny and polyvalent strategies tend to be more common in stressful environments (Huang et al. 2024). In addition, temperate phages can expand the metabolic repertoire of their bacterial hosts through auxiliary metabolic genes (AMGs), which may play a role in antibiotic resistance, virulence, biogeochemical nutrient cycling, and bacterial adaptation and evolution (Zimmerman et al. 2020; Chevallereau et al. 2022). Enrichment of AMGs associated with stress tolerance and xenobiotic degradation has been reported in contaminated environments (Huang et al. 2021; Zheng et al. 2022; Yuan et al. 2023). These genes could benefit the bacterial adaptive response and increase pollutant degradation; however, the role of phages in bioremediation still needs to be explored (Ru et al. 2023).

Bioaugmentation, which involves the inoculation of pollutant-degrading microbes, has been proposed as a promising solution for the remediation of contaminated ecosystems (El Fantroussi and Agathos 2005; Muter 2023). However, bacterial inoculants frequently fail to establish in situ, which reduces the reliability of inoculation technologies and success in the field (Kaminsky et al. 2019; Jurburg et al. 2022). An increase in the activity of lytic phage may limit the application of microbial-based technologies by reducing the survival of the inoculum, and consequently, the desired metabolic activity (Fu et al. 2009; Albright et al. 2022). Furthermore, the inoculation of allochthonous bacteria, which is a common strategy in biotechnological solutions, may be more affected by the phage community than native bacteria (Braga et al. 2020). However, the role of the phages in controlling inoculum abundance and functionality has received little attention.

In our previous work, we inoculated a consortium of polycyclic aromatic hydrocarbon (PAH)-degraders consisting of the two strains *Sphingobium* AM and *Paraburkholderia caledonica* Bk, into soils with different pollution exposure histories (Nieto et al. 2024). Inoculation did not increase PAH removal in the chronically contaminated soil, which was explained by the low survival of the inoculated strains due to predation by eukaryotic microorganisms. To further investigate the role of other potential predators in inoculum survival and functioning, this study characterizes the interaction between a phage and one strain during PAH-degradation at the laboratory scale for the first time. We isolated and characterised a phage from the chronically contaminated soil that infects *P. caledonica* Bk. This strain has the genomic potential to use PAHs as the sole carbon and energy source (Macchi et al. 2021; Nieto et al. 2023, 2025). However, it showed lower survival than strain AM in a chronically contaminated soil (Nieto et al. 2024). In addition, we investigated how different phage-bacteria ratios (i.e., the multiplicity of infection (MOI)) affect bacterial population dynamics and phenanthrene degradation at the laboratory scale. We hypothesised that a higher initial MOI would result in lower degradation efficiency due to higher host mortality.

## Materials and methods

### Screening of prophage and CRISPR-Cas arrays in the host genome

The genome of *Parabukholderia caledonica* Bk was previously sequenced (accession number: NHOM01; (Macchi et al. 2021)). We searched for prophage regions in the host genome using the PHASTEST web server (https://phastest.ca/) (Wishart et al. 2023). In addition, CRISPR (clustered regularly interspaced short palindromic repeats) arrays and their associated (Cas) proteins were detected using the CRISPRCasFinder web server (https://crisprcas.i2bc.paris-saclay.fr/CrisprCasFinder/Index) (Couvin et al. 2018).

### Phage enrichment, isolation, purification

A chronically contaminated soil (IPK) from a petrochemical plant in Ensenada, Argentina (34°53′19″S, 57° 55′ 38″W) was selected to isolate phages for the allochthonous PAH-degrading strain *P. caledonica* Bk. The IPK soil was previously treated by landfarming, with several applications of petrochemical sludge. Sampling was done approximately 10 years after the cessation of the petrochemical sludge treatments, showing a total PAH concentration of 573 ± 138 mg kg^−1^ dry soil (Cecotti et al. 2018; Festa et al. 2024).

For phage enrichment, 10 g of soil was mixed with 90 ml of LB broth supplemented with CaCl_2_ and CaSO­_4_ (1 mM) and 1 ml of an overnight culture of the Bk strain. After 24 h of incubation, 5 ml of each enrichment culture was centrifuged for 10 min at 3600 rpm, and the supernatant was filtered through a 0.22 µm nylon membrane. The filtrate was tested for phage activity against *P. caledonica* Bk using a double agar assay with 0.6% soft top agar. Plaques were collected with a sterile pipette tip and transferred to 200 µl of LB broth, incubated for 1 h at room temperature, and centrifuged for 10 min at 15,000 g at 4 °C. The supernatant was filtered (0.22 µm) and tested for phage activity. This process was repeated twice to ensure pure isolates.

### One-step growth curve

The one-step growth curve of the phage was determined by using a modified protocol previously described (Chen et al. 2020). Briefly, 5 ml of an overnight bacterial culture (OD_600_ of 0.3 to 0.5) was centrifuged at 8,000 rpm for 5 min. The cell pellets were resuspended in 500 µl of LB medium, and infected with 100 µl of phage suspension to yield a multiplicity of infection (MOI) of 0.01. After adsorption for 10 min at room temperature, the phage-host mixtures were centrifuged at 12,000 rpm for 10 min to remove unadsorbed phage particles. The cell pellets were resuspended in 5 ml of LB medium and incubated at 30 °C with constant shaking. Aliquots were collected every 20 min for up to 3 h and were immediately serially diluted. Phage titers were determined using the spotting plaque assay technique. Three independent replicates were performed for each assay.

### Killing curve

Cultures of *P. caledonica* Bk were infected at an early exponential phase (OD_600_ of 0.4) with different phage concentrations to obtain final MOIs of 0.001, 0.01, 0.1, and 1. Positive and negative controls were also prepared by excluding the phage or bacterial cells, respectively. After incubation in a microplate reader for 13 h at 30 °C, the OD_600_ of each well was measured at 30 min intervals after mixing of 10 s, with four replicates for each treatment.

### Thermal and pH stability assays

For the thermal stability test, 500 µl of filter-sterilised phage samples were incubated at 15°, 30°, 40°, 50°, 60° and 70 °C for 24 h. For the pH stability test, 40 µl of phage lysate were added to 3960 µl TM buffer with a pH ranging from 1 to 13 and incubated for 1 h and 24 h. After incubation, the phage titers were determined by a double-layer assay to determine the number of plaque-forming units.

### Phage DNA extraction, genome sequencing and assembly

The phage DNA was extracted from the lysates, as described by (Thurber et al. 2009). The isolated phage DNA was sequenced using the Oxford Nanopore MinION platform (MN45708). Library preparation followed the "Ligation Sequencing gDNA—Native Barcoding Kit 24 V14 Oxford" protocol (v. NBE_9169_v114_revH_15Sep2022) from Oxford Nanopore Technologies, with one modification: during the DNA repair step, incubation was extended to 15 min at 20 °C and 65 °C. Approximately 10 ng/µl of DNA was pooled to create a barcoded library with a final volume of ~ 50 µl, which onto an R10.4.1 (FLO-MIN114) flow cell in accordance with manufacturer instructions. Sequencing runs were conducted and monitored through the MinKNOW software. The raw signal data were first basecalled using Guppy basecaller (v. 6.5.7 + ca6d6af). The input fast5 files were processed using the high accuracy (HAC) basecalling model (dna_r10.4.1_e8.2_400bps_hac.cfg), optimized for the R10.4.1 flow cell chemistry.

Demultiplexing was conducted during the basecalling process using the barcoding scheme provided in the Native Barcoding Kit 24 (SQK-NBD114-24). To ensure all reads were retained for analysis, quality score filtering was disabled. To improve the accuracy of basecalling automatic calibration detection was enabled. Guppy was also employed for demultiplexing. Adapter sequences and other residual non-biological elements were trimmed using Porechop (v.0.2.3_seqan2.1.1) (Wick et al. 2017).

Genome assembly was performed using Flye (v.2.7-b1585) (Kolmogorov et al. 2019), which produced high-quality draft genomes with sufficient contiguity for subsequent error correction steps. The draft assemblies were further refined using Medaka (v.1.8.0) (Oxford Nanopore Technologies 2018). For prophage validation, CheckV (v.1.0.3) (Nayfach et al. 2021) and VirSorter (v.2.2.4) (Guo et al. 2021) were utilized. CheckV also assessed the quality and completeness of the prophage sequences. To assign taxonomic classifications, the assembled genomes were analyzed using GTDB-Tk (version v.2.3.2), a software tool based on the Genome Taxonomy Database (GTDB). Automatic annotation was performed using geNomad (v.1.7.1) and VIBRANT (v.1.2.1). Additionally, the unclassified proteins were manually classified using HHpred (probability > 0.9, e-value < 10^–5^) (Söding et al. 2005).

### Phylogenetic and comparative genomic analyses

To elucidate the taxonomy of *Paraburkholderia* phage IPK, most related phages were identified using the NCBI Virus database (https://www.ncbi.nlm.nih.gov/labs/virus/vssi/#/), vConTACT2 (Jang et al. 2019) and VipTree (https://www.genome.jp/viptree/) (Nishimura et al. 2017). Nine complete genomes were obtained from the NCBI dataset, and only two genomes were cluttered according to vContact2 (accession numbers: EU982300.1; OK665841.1). From the VipTree results, we selected 20 closely related phage genomes using the S_G_ score (Zhu et al. 2024). From these genomes, we constructed a phylogenetic tree using the Genome-BLAST Distance Phylogeny (GBDP) method in the Virus Classification and Tree Building Online Resource (VICTOR) (Meier-Kolthoff and Göker 2017). The resulting intergenomic distances were used to infer a balanced minimum evolution tree with branch support via FASTME including SPR postprocessing for the formulas D0. Additionally, intergenomic similarities were calculated using VIRIDIC (Moraru et al. 2020)**.**

### Effect of multiplicity of infection (MOI) on phenanthrene (PHN) degradation

To evaluate the effect of different phage-host ratios (i.e. multiplicity of infection (MOI)) during PHN degradation, *P. caledonica* Bk was grown in LB medium overnight at 30 °C and 150 rpm, centrifuged at 6000 rpm for 10 min, then washed three times with 0.85% NaCl and resuspended in the same solution. A density of 1*10^6^ CFU ml^−1^ was inoculated into 10 ml of Liquid Mineral Medium (LMM) (Vecchioli et al. 1990) supplemented with 200 mg l^−1^ of PHN as the sole carbon and energy source. Since no reduction in host OD was observed in the killing curve at the two lowest MOIs, we excluded the MOI 0.001, added a higher one and tested the following MOIs: 0.01, 0.1, 1, and 10. Bk cultures without phages were used as control. Each treatment was carried out by destructive triplicate monitoring at 0, 1, 2, 3 and 4 days of incubation at 30 °C and 150 rpm. Three consecutive chemical extractions with ethyl acetate were performed and PHN was measured by HPLC (Waters® XBridge C18 3.5 μm, 4.6 × 65 mm) following (Nieto et al. 2023)**.**

In parallel, triplicate cultures with the different MOIs were run and resampled at 0, 1, 2, 3 and 4 days of incubation to determine phage titers using the spotting plaque assay technique and bacterial abundances after plating on LB agar, and to describe virus-host-ratios over time. Day 4 phage samples were lost during processing, so they are excluded in the article.

### Statistical analysis

Statistical analyses were performed in R v 4.3.3 (R Core Team, 2024). Two-ways ANOVA were carried out to test the effects of MOI in degradation efficiency with MOI and time as independent variables, using the *rstatix* (v.0.7.2) (Kassambara 2023) R package. Shapiro and Levene tests were performed to check normality and homoscedasticity, respectively. Tukey’s Honestly Significant Difference (HSD) post-hoc test was used to perform pairwise comparisons between group means. Due to the lack of normality, significant differences between log_10_ of bacterial abundance across different MOIs over time were assessed using non-parametric Kruskal–Wallis test and Dunn test as a post-hoc test to perform pairwise comparisons between group means. PHN concentration, bacterial and phage counts are expressed as mean ± standard deviation.

## Results

### Screening of prophage and CRISPR-Cas arrays in the host genome

No prophage sequences were found in *P. caledonica* Bk. Three CRISPR elements that were not associated with *cas* genes were identified. The low level of evidence (level 1) indicates potentially invalid CRISPR arrays (Couvin et al. 2018).

### Biological characterization of phage

**Fig. 1:**
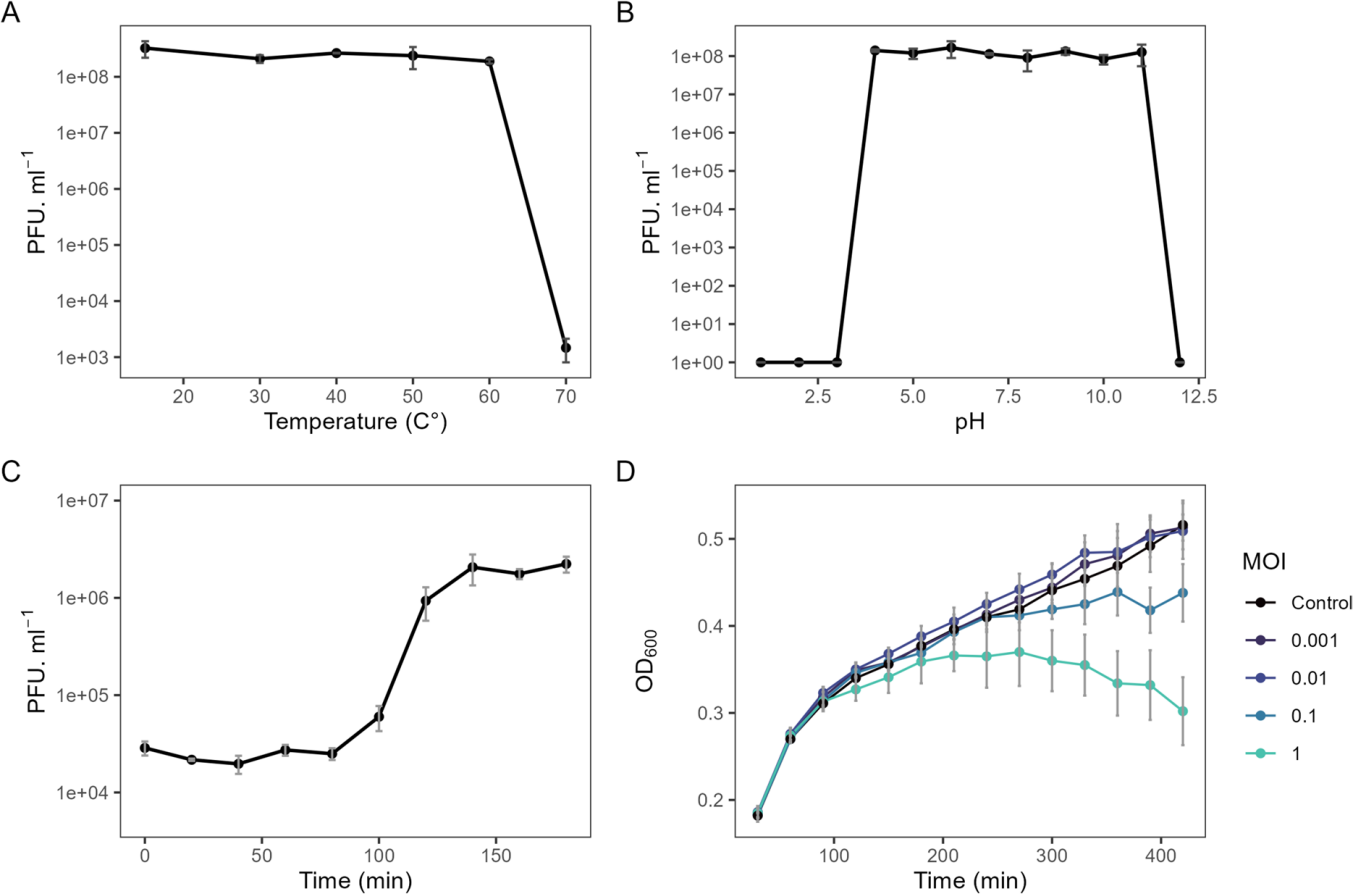
Biological characterization of phage IPK. **A** Thermostability profile. **B** pH stability profile. **C** One-step growth curve using an MOI of 0.01. **D** Killing curve at different MOI

One phage, *Paraburkholderia* phage IPK, was isolated from the chronically PAH-contaminated soil. Thermal and pH stability tests were performed and showed survival in a pH range of 4–11 and thermal stability up to 60 °C (Fig. 1A, B). The one-step growth curve showed a latent period of 80 min and a burst size of 80 PFU.cell^−1^ (Fig. 1C). The effect of the MOI on the survival of the host *P. caledonica* Bk was assessed using four MOIs (0.001, 0.01, 0.1 and 1). The lower MOIs did not inhibit host growth during the analysed period, whereas MOIs of 0.1 and 1 did. The highest MOI showed the largest inhibition, reducing growth within five hours of incubation (Fig. 1D).

### Phage genome characterization

**Fig. 2:**
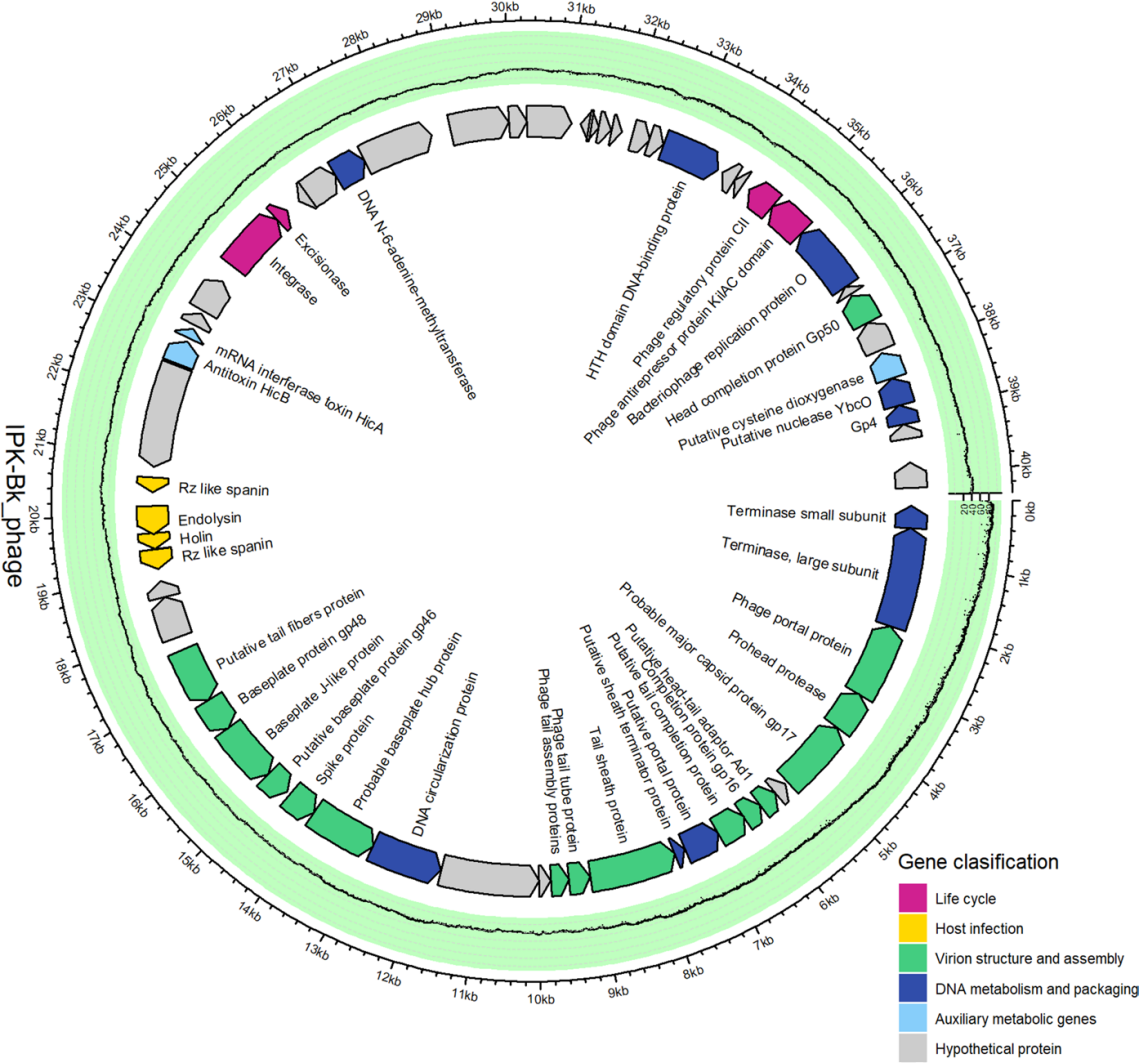
Genome overview of phage IPK. The different colours indicate different types of functional modules

Whole genome analysis showed that phage IPK is a dsDNA phage with a genome size of 40,356 bp and a GC content of 60.42%. A total of 63 ORFs were predicted, of which 43 were functionally annotated while 20 were annotated as hypothetical phage proteins (Fig. 2, Table S1). Around 25% were classified as structural proteins, including spike proteins, tail tube and tail sheath proteins, and putative major capsid proteins. Lifestyle prediction showed that the phage is temperate (99.98% probability). Among the ORFs related to life cycle, an integrase and an excisionase were annotated, which facilitate phage genome integration or excision into the host genome, respectively. In addition, an antirepressor protein KilAC and a phage regulatory protein CII were annotated, which play a role in the regulation of the lysogenic cycle. Three ORFs were annotated as auxiliary metabolic genes, including the antitoxin HicB and the mRNA interferase toxin HicA, which are involved in the regulation of bacterial growth, and a cysteine dioxygenase, which is involved in cysteine metabolism.

### Phylogenetic and comparative genome analysis

**Fig. 3:**
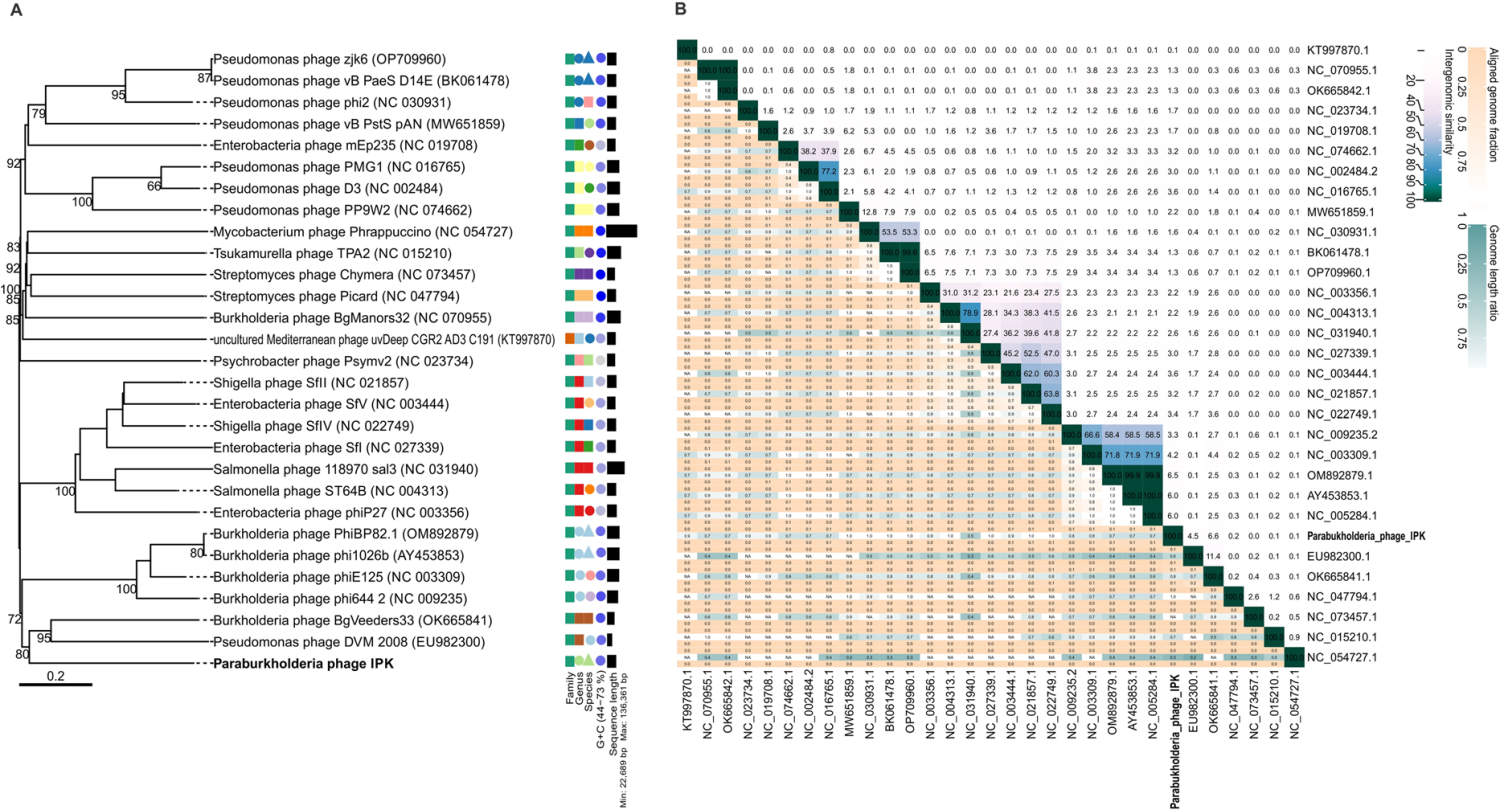
**A** Phylogenomic Genome-BLAST Distance Phylogeny (GBDP) method tree inferred using formula D0. The branch lengths of the resulting VICTOR trees are scaled according to the respective distance formula used. **B** Heatmap generated by VIRIDIC showing the intergenomic similarity values (right half) and alignment indicators (left half). The percent identity between two genomes was determined by BLASTn, integrating intergenomic similarity values with data on genome lengths and aligned genome fractions

Genomic analysis revealed that the phage IPK belongs to the class *Caudoviricetes*. A more detailed classification using VICTOR (Fig. 3A) revealed that phage IPK clustered with other *Burkholderia* and *Pseudomonas* phages and was more closely related to *Burkholderia* phage BgVeeders33 and *Pseudomonas* phage DVM 2008. The intergenomic similarity analysis showed low similarity with the selected genomes (Fig. 3B). Therefore, both analyses indicate that the phage IPK belongs to a new genus.

### Effects of phage density on phenanthrene biodegradation

**Fig. 4:**
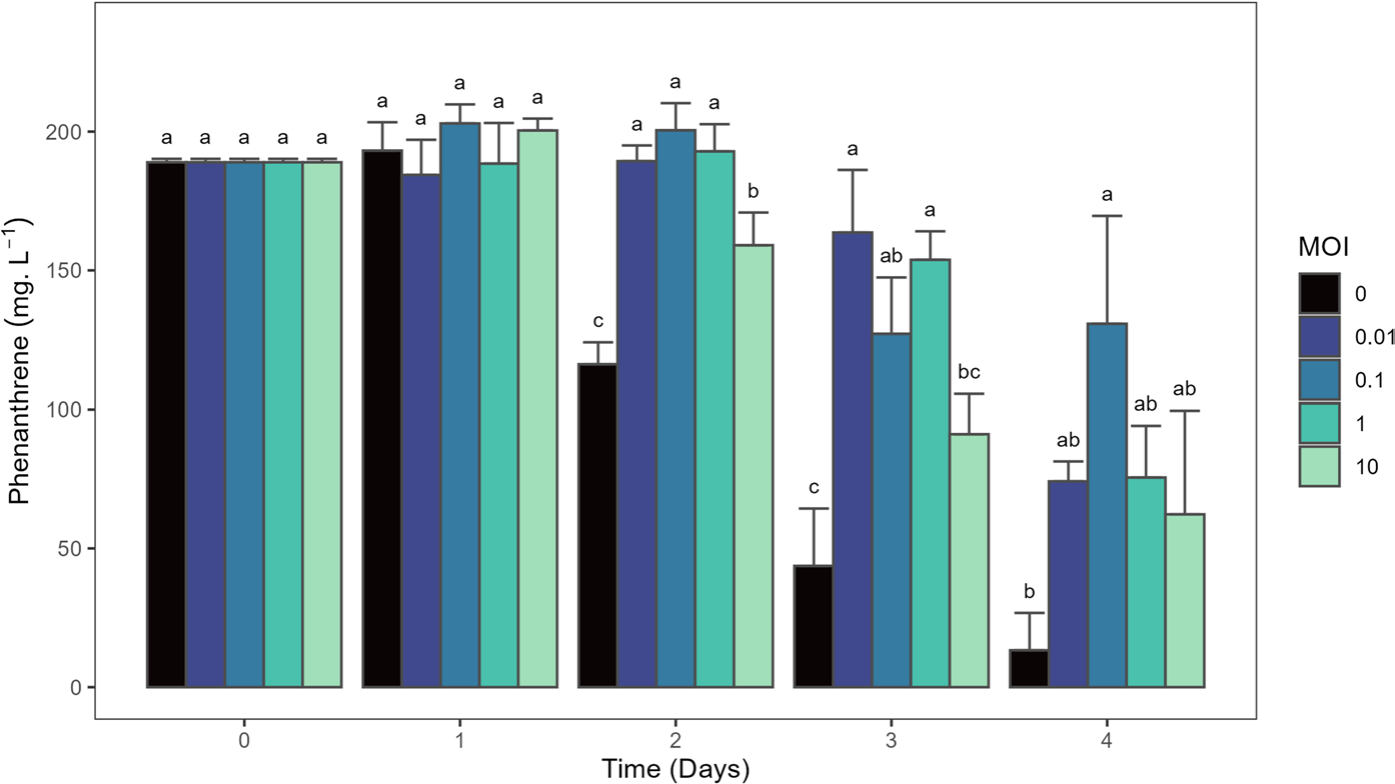
Concentration of phenanthrene (PHN) over time in *P. caledonica* Bk cultures at different MOI (0, 0.01, 0.1, 1 and 10) grown in PHN as only carbon and energy source (200 mg ml^−1^). The results are expressed as the mean values of independent triplicates and the error bars indicate standard deviation

The MOI affected the biodegradation efficiency of *P. caledonica* Bk (Fig. 4). A latency period was observed at all MOIs; however, its duration varied depending on the treatment. After two days of incubation, the control (MOI 0) and MOI 10 showed that 38.5 ± 4.2% and 15.8 ± 6.2% of PHN was degraded, respectively, while no degradation was observed at MOI 0.01, MOI 0.1 and MOI 1. On day three, the latter treatments showed 13.4 ± 11.9%, 32.7 ± 10.7% and 18.6 ± 5.4% of degradation, respectively, which were lower (p-value < 0.05) than the degradation observed at MOI 10 (51.8 ± 7.6%) and in the control (76.8 ± 10.9%). At the end of the incubation period, the highest and lowest degradation were observed in the control (93.0 ± 7.1%, p-value < 0.05) and at MOI 0.1 (30.8 ± 20.6%), respectively, while the other treatments showed intermediate degradation efficiencies.

**Fig. 5:**
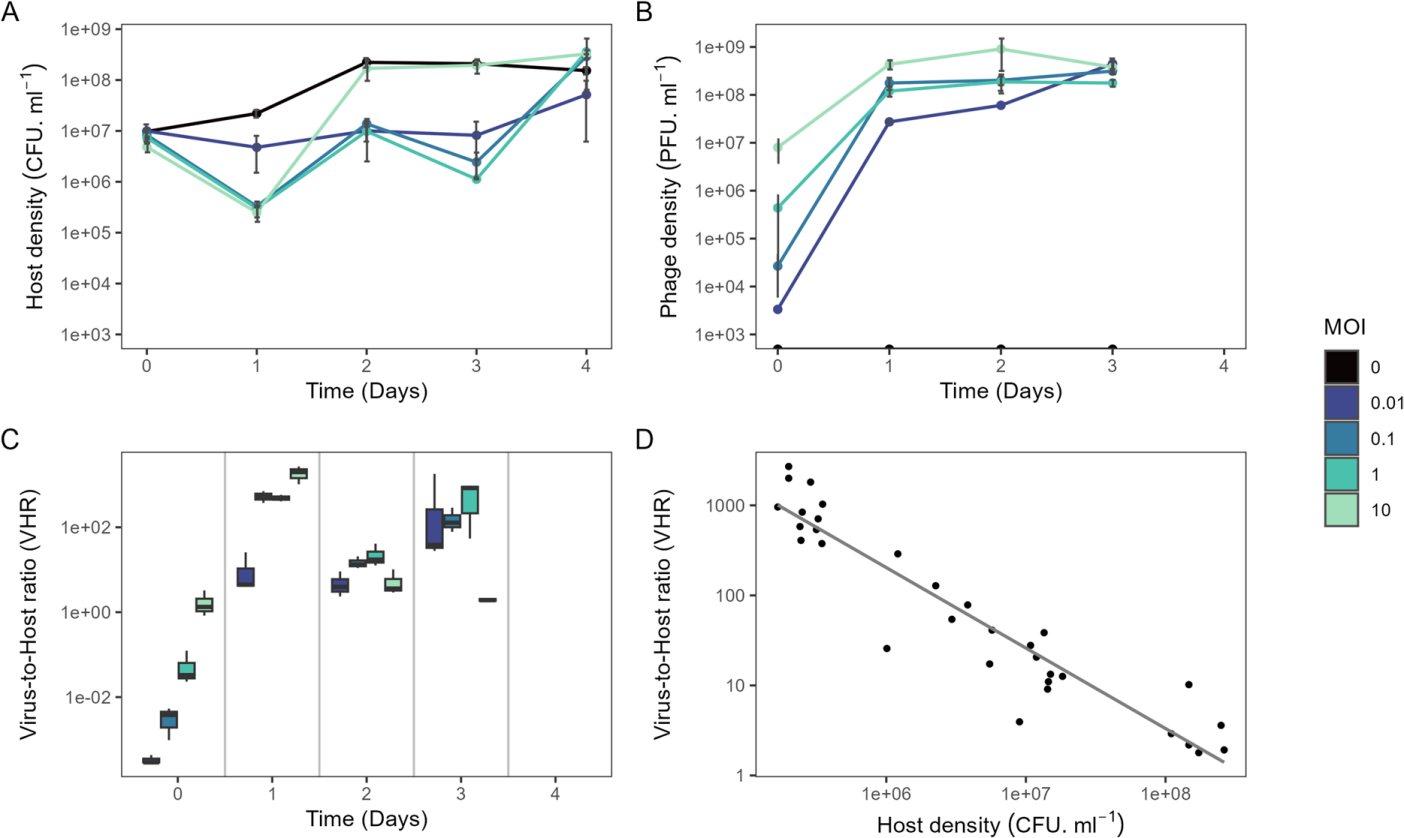
**A** bacterial, **B** phage counts and **C** Virus-host-ratios (VHR) over time in *P. caledonica* Bk cultures at different MOI (0, 0.01, 0.1, 1 and 10) grown in PHN as only carbon and energy source (200 mg ml^−1^). The results are expressed as the mean values of independent triplicates and the error bars indicate standard deviation. **D** Virus-to-host ratio as a function of host density

The population dynamics of the host and the phage were analysed during PHN degradation (Fig. 5A and B). The bacterial density in the control increased until day 2, reaching a density of 2.2*10^8^ ± 4.7*10^7^ CFU ml^−1^, which remained in the same range until the end of the incubation period (Fig. 5A). At MOI 0.01, the density of the host remained constant until the final day of the incubation period, when an increase was observed. Compared to the control, bacterial densities in the MOI 0.1, MOI 1 and MOI 10 treatments (*p* < 0.05) initially decreased after 1 day to values close to 10^5^ CFU ml^−1^. However, bacterial density in the MOI 10 treatment was the same as in the control at day 2 (~ 10^8^ CFU ml^−1^, *p* > 0.05), while the intermediate MOI treatments only reached similar bacterial abundances by the end of the incubation period at (Fig. 5A). Phage abundances increased in all treatments after one day of incubation, with MOI 0.1, MOI 1 and MOI 10 resulting in phage densities close to 10^8^ PFU ml^−1^(Fig. 5B). After three days all treatments showed similar PFU values (Fig. 5B).

Different virus-host-ratios over time were observed across the treatments (Fig. 5C). After three days of incubation, the MOI 10 treatment showed a ratio close to 1, whereas in the other treatments, the proportion of phages exceeded that of the bacterial host by two orders of magnitude. The differences between the treatments correlated with the increase in host density observed at MOI 10 at day 2 (Fig. 5A), while the number of PFU ml^−1^ remained constant (Fig. 5B). In addition, a negative correlation was observed between the log of the virus-host-ratio and host density (*R*^*2*^ = 0.89, p-value < 0.01, Fig. 5D).

## Discussion

Bioaugmentation is considered a promising and sustainable solution for the remediation of contaminated soils. However, the outcome of inoculation is variable, mainly due to the lack of establishment of the inoculum, which may limit its applicability (Kaminsky et al. 2019; Jurburg et al. 2022). After inoculation, the abundance of the inoculated bacteria can trigger potential predators, including phages, to become active, which may impact the survival of the inoculum (Shapiro and Kushmaro 2011; Albright et al. 2022). Although phages are one of the major causes of bacterial mortality, the ecological implications of phage-mediated top-down regulation of inoculants have received little attention. To our knowledge, only one previous study has reported a failure of bioaugmentation due to the lack of establishment of the inoculant as a consequence of phage activity (Fu et al. 2009).

In our previous study (Nieto et al. 2024), the inoculation of a PAH-degrading consortium consisting of the strains *Sphingobium* AM and *Paraburkholderia caledonica* Bk failed to remove PAHs in a chronically PAH-contaminated soil. We tracked the fate of the C^13^-labelled biomass of the consortium using DNA-SIP and identified a rapid response of the eukaryotic predator community after inoculation, which correlated with the low survival of the inoculated strains. However, this study did not assess the role of phages, which may also contribute to the observed low-inoculum survival. Here, we present a first description of the phage-host interaction during pollutant degradation. We achieved this by isolating a new phage from the same contaminated soil that infects one of the inoculated strains (*Paraburkholderia caledonica* Bk) and by characterising the effects of different phage-host ratios (i.e. MOIs) on this process under controlled laboratory conditions.

The genus *Paraburkholderia* includes mainly environmental species with promising biotechnological potential (Vio et al. 2020). Members of this genus show a high frequency of prophages in their genomes, which correlates with its diversification (Pratama et al. 2018). The Bk strain lacks any valid CRISPR-Cas arrays or prophage sequences in its genome. The presence of these elements in bacterial genomes confers immunity to the bacterial host against new infection (Hampton et al. 2020). CRISPR-Cas systems are adaptive immune mechanisms in which transcribed CRISPR spacers recognize matching sequences and direct Cas nucleases to these target sites during subsequent encounters with mobile genetic elements, leading to their inactivation (Koonin and Makarova 2019). Prophages generally encode systems that prevent DNA entry, inhibiting infection by other phages (Dy et al. 2014). The absence of these elements in *P. caledonica* Bk could indicate its susceptibility to phage infection (Obeng et al. 2016; Hooton et al. 2020).

Phylogenetic analysis suggested that the phage IPK belongs to a new genus related to *Burkholderia* and *Pseudomonas* prophages (DeShazer 2004; Mavrodi et al. 2009; Khrongsee et al. 2024) (Fig. 3). The presence of lysogeny-related genes indicates that the isolated phage is a temperate phage. Lysogeny may represent an adaptive strategy for phages to cope with adverse environments (Huang et al. 2024). Some recent studies have shown that the number of prophages correlates with contamination severity (Huang et al. 2021; Zheng et al. 2022; Yuan et al. 2023). However, Xia et al. (2023) reported that there was a higher proportion of lysogenic phages at low benzo[a]pyrene exposure than at high exposure. Despite variable phage-bacterium interactions, AMGs encoded by phages and associated with microbial antioxidant and pollutant degradation were enriched in these contaminated environments. This suggests that phages may play a role in the adaptive response of the host by increasing the biodegradation potential (Zheng et al. 2022; Xia et al. 2023). The genome of phage IPK contains three AMGs, including a cysteine dioxygenase, the antitoxin HicB and the mRNA interferase toxin HicA (Fig. 2, Table S1). Xia et al (2023) reported that the AMGs potentially involved in acid metabolism were the most abundant AMGs in lysogenic phages under benzo[a]pyrene exposure, and showed the expression of these AMGs at low exposure levels. In addition, the toxin-antitoxin system HicAB contributes to regulating bacterial growth under stress conditions and maintaining the prophage in the host (Qian et al. 2022; Encina-Robles et al. 2024). Therefore, these AMGs may expand the metabolic profile of the host and enhance the adaptability of microbes to the environment (Huang et al. 2021). Some studies have reported the expression of AMGs and functional analysis of the expressed proteins (Wu et al. 2022; Xia et al. 2023), however, there remains a need to investigate how these genes contribute to bioremediation processes.

The relationship between phage activity and hydrocarbon degradation is not well understood (Ru et al. 2023). Our results showed that the phage-host dynamics affected the PHN degradation efficiency of the Bk strain (Fig. 4). Phage-host interactions are dynamic and different processes act on different time scales. Arms-race dynamics dominate over short timescales, while fluctuating selection dynamics characterize long-term interactions, enabling the coexistence of resistant and susceptible bacterial types (Guerrero et al. 2021; Koskella et al. 2022). Due to the nature of our experimental setup, in which the PHN is quickly degraded, we could not assess long-term dynamics which could have resulted in overlooking the codiversification processes between phage and host (Koskella et al 2022). Phages can respond rapidly following inoculum introduction, impacting its survival (Pantastico-Caldas et al. 1992). Given that the first few days after inoculation are critical for success (Van Dyke and Prosser 2000), it is essential to characterize these early dynamics for optimizing soil inoculation strategies. Differences in the short-term dynamics were observed among the treatments. All treatments showed a lag period in PHN degradation, including the control without phages as previously reported (Nieto et al. 2023) the duration of this period differed between the treatments and correlated with host bacterial density (Fig. 5A). After one day, all treatments with an MOI ≥ 0.1 showed a strong reduction in host density. Notably, only the MOI 10 treatment resulted in a rapid host recovery by day two, reaching densities comparable to the control, which coincided with an increase in PHN degradation. In contrast, lower and intermediate MOIs (0.01–1) led to sustained viral amplification and prolonged suppression of the host, delaying recovery and PHN degradation until day three. The degradation latency in Bk has been linked to a delay in the expression of PAH-degrading catabolic genes (Nieto et al. 2023). Other studies have suggested a density-dependent regulation of these genes via quorum sensing in different microbes (Yong and Zhong 2013; Yu et al. 2020). Since phages affect host density, they consequently influence the quorum sensing response and host functioning (Coolahan and Whalen 2025). These findings suggest that the initial MOI shapes the host–phage interaction, leading to either a transient burst or prolonged suppression of the host population, which, in turn, could modulate biodegradation outcomes.

Based on these results we reject our hypothesis that a higher initial MOI would result in lower degradation efficiency. Higher phage abundance has been shown to lead to a higher selective pressure for resistant forms and may contribute to an increase in host abundance (Koskella and Brockhurst 2014). However, similar mutation rates should be expected across the treatments and these would not explain the fast recovery observed only at MOI 10. In addition, nutrient release due to viral shunt has been linked with enhanced hydrocarbon degradation performance (Rosenberg et al. 2010). However, we observed similar mortality in the three treatments with higher MOI, which likely indicates similar nutrient release, and therefore does not explain the difference we observed. We speculate that an increase in lysogeny, the frequency of which has been shown to correlate with coinfection rates and higher MOI (Herskowitz and Hagen 1980), and which may confer immunity to the host (Hampton et al. 2020), may be a potential mechanism responsible for the rapid recovery and PHN degradation observed at MOI 10. The presence of lysogenic ORFs in phage IPK, in addition to its phylogenetic relationship with other prophages, supports this assumption. We also observed a negative correlation between virus-to-host ratio and bacterial abundance (Fig. 5D), which is consistent with the previously described pattern both at genus (Coutinho et al. 2017) and community levels (Knowles et al. 2016; Silveira et al. 2021), and which aligns with the Piggyback-the-Winner model (Knowles et al. 2016). Further experiments testing the expression of lysogenic markers or the lysogenization (Riley et al. 2012; Ruiz-Cruz et al. 2020) of surviving hosts under varying MOI conditions (Luque and Silveira 2020) are needed to confirm our interpretation.

The role of phages in host functionality, particularly in polluted environments, remains largely overlooked. Laboratory models continue to be valuable tools for gaining new insights into the complexity of these interactions (Puxty and Millard 2023), which could be crucial for optimising inoculum screening and application. The initial phage-host ratio exerts a strong influence on both bacterial survival and degradation efficiency. High initial phage pressure can result in substantial bacterial mortality; however, over time, nutrients released by lysis may support bacterial regrowth (Rosenberg et al. 2010) and accelerate degradation. Interestingly, under elevated phage pressure, the potential for lysogeny may confer a selective advantage by stabilizing bacterial populations and providing host-immunity against superinfection (Paul 2008; Weitz et al. 2013); however, it remains to be studied how increased rate of lysogeny may affect the potential for horizontal gene transfer in this context (Howard-Varona et al. 2017; Molina-Quiroz et al. 2020). As inoculum survival is pivotal for bioaugmentation success (Albright et al. 2022), conferring resistance to phage infection can increase the probability of inoculum establishment. The identification of strains that possess defense mechanisms, such as CRISPR-Cas systems, could facilitate the selection of more robust candidates. However, phages have evolved counter-strategies, and infection after inoculation remains likely (Koskella and Brockhurst 2014). Inoculum size has been demonstrated to be a significant factor affecting the survival of introduced microbes (Kaminsky et al. 2019); however the relationship between phages and inoculum in soils remains largely unexplored. The spatial structuring of soils could also have an effect on phage-host dynamics by limiting phage dispersal and promoting coexistence (Koskella et al 2022). Gaining a better understanding of the lysogenic–lytic switch could prove instrumental in modulating this interaction (Voigt et al 2021), enhancing resistance to phage infection, and ultimately improving inoculum survival and the success of bioaugmentation strategies.

## Conclusion

The findings of our study demonstrate the impact of phage-host ratios on the degradation performance of *P. caledonica* Bk, which correlated with host abundance. Notably, the treatment with the highest initial MOIs showed faster degradation compared to the treatment with lower initial MOIs. Although this interaction and the observed dynamics may change in a complex, structured system such as soil (Koskella et al. 2022), these results underscore the need to consider the role of phage-host interactions on inoculum survival and its function (e.g. degradation efficiency), which could be key to improving inoculation success. These findings underscore the importance of further research to investigate the complex processes related to temperate phages, including auxiliary metabolic genes, lysogenic marker expression, complex matrices, and long-term dynamics, in order to advance biotechnological solutions.

## Supplementary Information

Below is the link to the electronic supplementary material.

Supplementary file1 (DOCX 28 KB)

## Data Availability

The *Paraburkholderia* phage IPK genome sequence was deposited in NCBI under the accession number PV588664.

## References

[CR1] Albright MBN, Louca S, Winkler DE et al (2022) Solutions in microbiome engineering: prioritizing barriers to organism establishment. ISME J 16:331–338. 10.1038/s41396-021-01088-5 34420034 10.1038/s41396-021-01088-5 PMC8776856

[CR2] Braga LPP, Spor A, Kot W et al (2020) Impact of phages on soil bacterial communities and nitrogen availability under different assembly scenarios. Microbiome 8:52. 10.1186/s40168-020-00822-z 32252805 10.1186/s40168-020-00822-z PMC7137350

[CR3] Brown TL, Charity OJ, Adriaenssens EM (2022) Ecological and functional roles of bacteriophages in contrasting environments: marine, terrestrial and human gut. Curr Opin Microbiol 70:102229. 10.1016/j.mib.2022.102229 36347213 10.1016/j.mib.2022.102229

[CR4] Carreira C, Lønborg C, Acharya B et al (2024) Integrating viruses into soil food web biogeochemistry. Nat Microbiol 9:1918–1928. 10.1038/s41564-024-01767-x 39095499 10.1038/s41564-024-01767-x

[CR5] Cecotti M, Coppotelli BM, Mora VC et al (2018) Efficiency of surfactant-enhanced bioremediation of aged polycyclic aromatic hydrocarbon-contaminated soil: link with bioavailability and the dynamics of the bacterial community. Sci Total Environ 634:224–234. 10.1016/j.scitotenv.2018.03.303 29627545 10.1016/j.scitotenv.2018.03.303

[CR6] Chen L, Liu Q, Fan J et al (2020) Characterization and genomic analysis of ValSw3-3, a new Siphoviridae bacteriophage infecting *Vibrio alginolyticus*. J Virol. 10.1128/JVI.00066-20 32132234 10.1128/JVI.00066-20 PMC7199398

[CR7] Chevallereau A, Pons BJ, van Houte S, Westra ER (2022) Interactions between bacterial and phage communities in natural environments. Nat Rev Microbiol 20:49–62. 10.1038/s41579-021-00602-y 34373631 10.1038/s41579-021-00602-y

[CR8] Coolahan M, Whalen KE (2025) A review of quorum-sensing and its role in mediating interkingdom interactions in the ocean. Commun Biol 8:179. 10.1038/s42003-025-07608-9 39905218 10.1038/s42003-025-07608-9 PMC11794697

[CR9] Coutinho FH, Silveira CB, Gregoracci GB et al (2017) Marine viruses discovered via metagenomics shed light on viral strategies throughout the oceans. Nat Commun 8:15955. 10.1038/ncomms15955 28677677 10.1038/ncomms15955 PMC5504273

[CR10] Couvin D, Bernheim A, Toffano-Nioche C et al (2018) Crisprcasfinder, an update of crisrfinder, includes a portable version, enhanced performance and integrates search for Cas proteins. Nucleic Acids Res 46:W246–W251. 10.1093/nar/gky425 29790974 10.1093/nar/gky425 PMC6030898

[CR11] DeShazer D (2004) Genomic diversity of *Burkholderia pseudomallei* clinical isolates: subtractive hybridization reveals a *Burkholderia mallei*-specific prophage in *B. pseudomallei* 1026b. J Bacteriol 186:3938–3950. 10.1128/JB.186.12.3938-3950.2004 15175308 10.1128/JB.186.12.3938-3950.2004 PMC419931

[CR12] Dy RL, Richter C, Salmond GPC, Fineran PC (2014) Remarkable mechanisms in microbes to resist phage infections. Annu Rev Virol 1:307–331. 10.1146/annurev-virology-031413-085500 26958724 10.1146/annurev-virology-031413-085500

[CR13] El Fantroussi S, Agathos SN (2005) Is bioaugmentation a feasible strategy for pollutant removal and site remediation? Curr Opin Microbiol 8:268–275. 10.1016/j.mib.2005.04.011 15939349 10.1016/j.mib.2005.04.011

[CR14] Encina-Robles J, Pérez-Villalobos V, Bustamante P (2024) The HicAB system: characteristics and biological roles of an underappreciated toxin-antitoxin system. Int J Mol Sci. 10.3390/ijms252212165 39596231 10.3390/ijms252212165 PMC11594946

[CR15] Festa S, Nieto E, Raposeiras Aldorino P et al (2024) Complementing culture-dependent and -independent approaches is essential when assessing bacterial community potential functions in chronically PAH-contaminated soil. Pedosphere. 10.1016/j.pedsph.2024.08.005

[CR16] Fu S, Fan H, Liu S et al (2009) A bioaugmentation failure caused by phage infection and weak biofilm formation ability. J Environ Sci (China) 21:1153–1161. 10.1016/S1001-0742(08)62396-7 19862932 10.1016/s1001-0742(08)62396-7

[CR17] Guerrero LD, Pérez MV, Orellana E et al (2021) Long-run bacteria-phage coexistence dynamics under natural habitat conditions in an environmental biotechnology system. ISME J 15:636–648. 10.1038/s41396-020-00802-z 33067586 10.1038/s41396-020-00802-z PMC8027832

[CR18] Guo J, Bolduc B, Zayed AA et al (2021) Virsorter2: a multi-classifier, expert-guided approach to detect diverse DNA and RNA viruses. Microbiome 9:37. 10.1186/s40168-020-00990-y 33522966 10.1186/s40168-020-00990-y PMC7852108

[CR19] Hampton HG, Watson BNJ, Fineran PC (2020) The arms race between bacteria and their phage foes. Nature 577:327–336. 10.1038/s41586-019-1894-8 31942051 10.1038/s41586-019-1894-8

[CR20] Herskowitz I, Hagen D (1980) The lysis-lysogeny decision of phage lambda: explicit programming and responsiveness. Annu Rev Genet 14:399–445. 10.1146/annurev.ge.14.120180.002151 6452089 10.1146/annurev.ge.14.120180.002151

[CR21] Hooton S, D’Angelantonio D, Hu Y et al (2020) Campylobacter bacteriophage DA10: an excised temperate bacteriophage targeted by CRISPR-cas. BMC Genomics 21:400. 10.1186/s12864-020-06808-3 32532247 10.1186/s12864-020-06808-3 PMC7291426

[CR22] Howard-Varona C, Hargreaves KR, Abedon ST, Sullivan MB (2017) Lysogeny in nature: mechanisms, impact and ecology of temperate phages. ISME J 11:1511–1520. 10.1038/ismej.2017.16 28291233 10.1038/ismej.2017.16 PMC5520141

[CR23] Huang D, Yu P, Ye M et al (2021) Enhanced mutualistic symbiosis between soil phages and bacteria with elevated chromium-induced environmental stress. Microbiome 9:150. 10.1186/s40168-021-01074-1 34183048 10.1186/s40168-021-01074-1 PMC8240259

[CR24] Huang D, Xia R, Chen C et al (2024) Adaptive strategies and ecological roles of phages in habitats under physicochemical stress. Trends Microbiol 32:902–916. 10.1016/j.tim.2024.02.002 38433027 10.1016/j.tim.2024.02.002

[CR25] Jang B, Bolduc B, Zablocki O et al (2019) Taxonomic assignment of uncultivated prokaryotic virus genomes is enabled by gene-sharing networks. Nat Biotechnol 37:632–639. 10.1038/s41587-019-0100-8 31061483 10.1038/s41587-019-0100-8

[CR26] Jurburg SD, Eisenhauer N, Buscot F et al (2022) Potential of microbiome-based solutions for agrifood systems. Nat Food 3:557–560. 10.1038/s43016-022-00576-x 37118595 10.1038/s43016-022-00576-x

[CR27] Jurburg SD, Hom EFY, Chatzinotas A (2023) Beyond pathogenesis: detecting the full spectrum of ecological interactions in the virosphere. PLoS Biol 21:e3002109. 10.1371/journal.pbio.3002109 37186573 10.1371/journal.pbio.3002109 PMC10184920

[CR28] Kaminsky LM, Trexler RV, Malik RJ et al (2019) The inherent conflicts in developing soil microbial inoculants. Trends Biotechnol 37:140–151. 10.1016/j.tibtech.2018.11.011 30587413 10.1016/j.tibtech.2018.11.011

[CR29] Kassambara A (2023) Pipe-friendly framework for basic statistical tests • rstatix. https://rpkgs.datanovia.com/rstatix/. Accessed 23 Jun 2024

[CR30] Khrongsee P, Irby I, Akaphan P et al (2024) A comprehensive study of prophage islands in *Burkholderia pseudomallei* complex. Front Bacteriol. 10.3389/fbrio.2024.1339809

[CR31] Knowles B, Silveira CB, Bailey BA et al (2016) Lytic to temperate switching of viral communities. Nature 531:466–470. 10.1038/nature17193 26982729 10.1038/nature17193

[CR32] Kolmogorov M, Yuan J, Lin Y, Pevzner PA (2019) Assembly of long, error-prone reads using repeat graphs. Nat Biotechnol 37:540–546. 10.1038/s41587-019-0072-8 30936562 10.1038/s41587-019-0072-8

[CR33] Koonin EV, Makarova KS (2019) Origins and evolution of CRISPR-Cas systems. Philos Trans R Soc Lond B Biol Sci 374:20180087. 10.1098/rstb.2018.0087 30905284 10.1098/rstb.2018.0087 PMC6452270

[CR34] Koskella B, Brockhurst MA (2014) Bacteria-phage coevolution as a driver of ecological and evolutionary processes in microbial communities. FEMS Microbiol Rev 38:916–931. 10.1111/1574-6976.12072 24617569 10.1111/1574-6976.12072 PMC4257071

[CR35] Koskella B, Hernandez CA, Wheatley RM (2022) Understanding the impacts of bacteriophage viruses: from laboratory evolution to natural ecosystems. Annu Rev Virol 9:57–78. 10.1146/annurev-virology-091919-075914 35584889 10.1146/annurev-virology-091919-075914

[CR36] Kuzyakov Y, Mason-Jones K (2018) Viruses in soil: nano-scale undead drivers of microbial life, biogeochemical turnover and ecosystem functions. Soil Biol Biochem 127:305–317. 10.1016/j.soilbio.2018.09.032

[CR37] Luque A, Silveira CB (2020) Quantification of lysogeny caused by phage coinfections in microbial communities from biophysical principles. mSystems. 10.1128/mSystems.00353-20 32934113 10.1128/mSystems.00353-20 PMC7498681

[CR38] Macchi M, Festa S, Nieto E et al (2021) Design and evaluation of synthetic bacterial consortia for optimized phenanthrene degradation through the integration of genomics and shotgun proteomics. Biotechnol Rep 29:e00588. 10.1016/j.btre.2021.e00588 10.1016/j.btre.2021.e00588 PMC7809168 33489789

[CR39] Mäntynen S, Laanto E, Oksanen HM et al (2021) Black box of phage-bacterium interactions: exploring alternative phage infection strategies. Open Biol 11:210188. 10.1098/rsob.210188 34520699 10.1098/rsob.210188 PMC8440029

[CR40] Mavrodi DV, Loper JE, Paulsen IT, Thomashow LS (2009) Mobile genetic elements in the genome of the beneficial rhizobacterium *Pseudomonas fluorescens* Pf-5. BMC Microbiol 9:8. 10.1186/1471-2180-9-8 19144133 10.1186/1471-2180-9-8 PMC2647930

[CR41] Meier-Kolthoff JP, Göker M (2017) VICTOR: genome-based phylogeny and classification of prokaryotic viruses. Bioinformatics 33:3396–3404. 10.1093/bioinformatics/btx440 29036289 10.1093/bioinformatics/btx440 PMC5860169

[CR42] Molina-Quiroz RC, Dalia TN, Camilli A et al (2020) Prophage-dependent neighbor predation fosters horizontal gene transfer by natural transformation. mSphere. 10.1128/mSphere.00975-20 33177216 10.1128/mSphere.00975-20 PMC7657591

[CR43] Moraru C, Varsani A, Kropinski AM (2020) VIRIDIC-a novel tool to calculate the intergenomic similarities of prokaryote-infecting viruses. Viruses 12:111268. 10.3390/v12111268 10.3390/v12111268 PMC7694805 33172115

[CR44] Muter O (2023) Current trends in bioaugmentation tools for bioremediation: a critical review of advances and knowledge gaps. Microorganisms. 10.3390/microorganisms11030710 36985282 10.3390/microorganisms11030710 PMC10056695

[CR45] Nayfach S, Camargo AP, Schulz F et al (2021) Checkv assesses the quality and completeness of metagenome-assembled viral genomes. Nat Biotechnol 39:578–585. 10.1038/s41587-020-00774-7 33349699 10.1038/s41587-020-00774-7 PMC8116208

[CR46] Nieto EE, Macchi M, Valacco MP et al (2023) Metaproteomic and gene expression analysis of interspecies interactions in a PAH-degrading synthetic microbial consortium constructed with the key microbes of a natural consortium. Biodegradation 34:181–197. 10.1007/s10532-022-10012-3 36596914 10.1007/s10532-022-10012-3

[CR47] Nieto EE, Jurburg SD, Steinbach N et al (2024) DNA stable isotope probing reveals the impact of trophic interactions on bioaugmentation of soils with different pollution histories. Microbiome 12:146. 10.1186/s40168-024-01865-2 39113100 10.1186/s40168-024-01865-2 PMC11305082

[CR48] Nieto EE, Festa S, Colman D et al (2025) Challenging the impact of consortium diversity on bioaugmentation efficiency and native bacterial community structure in an acutely PAH-contaminated soil. Environ Sci Pollut Res Int 32:5589–5604. 10.1007/s11356-025-35987-3 39939570 10.1007/s11356-025-35987-3

[CR49] Nishimura Y, Yoshida T, Kuronishi M et al (2017) Viptree: the viral proteomic tree server. Bioinformatics 33:2379–2380. 10.1093/bioinformatics/btx157 28379287 10.1093/bioinformatics/btx157

[CR50] Obeng N, Pratama AA, Elsas JD (2016) The significance of mutualistic phages for bacterial ecology and evolution. Trends Microbiol 24:440–449. 10.1016/j.tim.2015.12.009 26826796 10.1016/j.tim.2015.12.009

[CR51] Oxford Nanopore Technologies (2018) Medaka. Version 1.8.0. GitHub

[CR52] Pantastico-Caldas M, Duncan KE, Istock CA, Bell JA (1992) Population dynamics of bacteriophage and *bacillus subtilis* in soil. Ecology 73:1888–1902. 10.2307/1940040

[CR53] Paul JH (2008) Prophages in marine bacteria: dangerous molecular time bombs or the key to survival in the seas? ISME J 2:579–589. 10.1038/ismej.2008.35 18521076 10.1038/ismej.2008.35

[CR54] Pratama AA, Chaib De Mares M, van Elsas JD (2018) Evolutionary history of bacteriophages in the genus paraburkholderia. Front Microbiol 9:835. 10.3389/fmicb.2018.00835 29867788 10.3389/fmicb.2018.00835 PMC5968390

[CR55] Puxty RJ, Millard AD (2023) Functional ecology of bacteriophages in the environment. Curr Opin Microbiol 71:102245. 10.1016/j.mib.2022.102245 36512900 10.1016/j.mib.2022.102245

[CR56] Qian C, Ma J, Liang J et al (2022) Comprehensive deciphering prophages in genus *Acetobacter* on the ecology, genomic features, toxin-antitoxin system, and linkage with CRISPR-Cas system. Front Microbiol 13:951030. 10.3389/fmicb.2022.951030 35983328 10.3389/fmicb.2022.951030 PMC9379143

[CR57] Riley LM, Veses-Garcia M, Hillman JD et al (2012) Identification of genes expressed in cultures of *E. coli* lysogens carrying the Shiga toxin-encoding prophage Φ24B. BMC Microbiol 12:42. 10.1186/1471-2180-12-42 22439817 10.1186/1471-2180-12-42 PMC3342100

[CR153] R Core Team (2024) R: A Language and environment for statistical computing. R foundation for statistical computing, Vienna, Austria. https://www.R-project.org/

[CR58] Rosenberg E, Bittan-Banin G, Sharon G et al (2010) The phage-driven microbial loop in petroleum bioremediation. Microb Biotechnol 3:467–472. 10.1111/j.1751-7915.2010.00182.x 21255344 10.1111/j.1751-7915.2010.00182.x PMC3815812

[CR59] Ru J, Xue J, Sun J et al (2023) Unveiling the hidden role of aquatic viruses in hydrocarbon pollution bioremediation. J Hazard Mater 459:132299. 10.1016/j.jhazmat.2023.132299 37597386 10.1016/j.jhazmat.2023.132299

[CR60] Ruiz-Cruz S, Parlindungan E, Erazo Garzon A et al (2020) Lysogenization of a lactococcal host with three distinct temperate phages provides homologous and heterologous phage resistance. Microorganisms. 10.3390/microorganisms8111685 33138325 10.3390/microorganisms8111685 PMC7693887

[CR61] Shapiro OH, Kushmaro A (2011) Bacteriophage ecology in environmental biotechnology processes. Curr Opin Biotechnol 22:449–455. 10.1016/j.copbio.2011.01.012 21354780 10.1016/j.copbio.2011.01.012

[CR62] Silveira CB, Luque A, Rohwer F (2021) The landscape of lysogeny across microbial community density, diversity and energetics. Environ Microbiol 23:4098–4111. 10.1111/1462-2920.15640 34121301 10.1111/1462-2920.15640

[CR63] Söding J, Biegert A, Lupas AN (2005) The HHpred interactive server for protein homology detection and structure prediction. Nucleic Acids Res 33:W244–W248. 10.1093/nar/gki408 15980461 10.1093/nar/gki408 PMC1160169

[CR64] Thingstad TF (2000) Elements of a theory for the mechanisms controlling abundance, diversity, and biogeochemical role of lytic bacterial viruses in aquatic systems. Limnol Oceanogr 45:1320–1328. 10.4319/lo.2000.45.6.1320

[CR65] Thurber RV, Haynes M, Breitbart M et al (2009) Laboratory procedures to generate viral metagenomes. Nat Protoc 4:470–483. 10.1038/nprot.2009.10 19300441 10.1038/nprot.2009.10

[CR66] Van Dyke MI, Prosser JI (2000) Enhanced survival of *Pseudomonas fluorescens* in soil following establishment of inoculum in a sterile soil carrier. Soil Biol Biochem 32:1377–1382. 10.1016/S0038-0717(00)00055-9

[CR67] Vecchioli GI, Del Panno MT, Painceira MT (1990) Use of selected autochthonous soil bacteria to enhanced degradation of hydrocarbons in soil. Environ Pollut 67:249–258. 10.1016/0269-7491(90)90190-n 15092212 10.1016/0269-7491(90)90190-n

[CR68] Vio SA, García SS, Casajus V et al (2020) Paraburkholderia. Beneficial microbes in agro-ecology. Elsevier, Amsterdam, pp 271–311

[CR69] Voigt E, Rall BC, Chatzinotas A et al (2021) Phage strategies facilitate bacterial coexistence under environmental variability. PeerJ 9:e12194. 10.7717/peerj.12194 34760346 10.7717/peerj.12194 PMC8572521

[CR70] Weitz JS, Poisot T, Meyer JR et al (2013) Phage-bacteria infection networks. Trends Microbiol 21:82–91. 10.1016/j.tim.2012.11.003 23245704 10.1016/j.tim.2012.11.003

[CR71] Wick RR, Judd LM, Holt KE (2017) Porechop: adapter trimming tool for Oxford Nanopore reads. Version 0.2.3

[CR72] Wishart DS, Han S, Saha S et al (2023) PHASTEST: faster than PHASTER, better than PHAST. Nucleic Acids Res 51:W443–W450. 10.1093/nar/gkad382 37194694 10.1093/nar/gkad382 PMC10320120

[CR73] Wu R, Smith CA, Buchko GW et al (2022) Structural characterization of a soil viral auxiliary metabolic gene product - a functional chitosanase. Nat Commun 13:5485. 10.1038/s41467-022-32993-8 36123347 10.1038/s41467-022-32993-8 PMC9485262

[CR74] Xia R, Sun M, Balcázar JL et al (2023) Benzo[a]pyrene stress impacts adaptive strategies and ecological functions of earthworm intestinal viromes. ISME J 17:1004–1014. 10.1038/s41396-023-01408-x 37069233 10.1038/s41396-023-01408-x PMC10284932

[CR75] Yong Y-C, Zhong J-J (2013) Regulation of aromatics biodegradation by rhl quorum sensing system through induction of catechol meta-cleavage pathway. Bioresour Technol 136:761–765. 10.1016/j.biortech.2013.03.134 23582222 10.1016/j.biortech.2013.03.134

[CR76] Yu Z, Hu Z, Xu Q et al (2020) The LuxI/LuxR-type quorum sensing system regulates degradation of polycyclic aromatic hydrocarbons via two mechanisms. Int J Mol Sci. 10.3390/ijms21155548 32756387 10.3390/ijms21155548 PMC7432010

[CR77] Yuan S, Friman V-P, Balcazar JL et al (2023) Viral and bacterial communities collaborate through complementary assembly processes in soil to survive organochlorine contamination. Appl Environ Microbiol 89:e0181022. 10.1128/aem.01810-22 36809072 10.1128/aem.01810-22 PMC10056961

[CR78] Zheng X, Jahn MT, Sun M et al (2022) Organochlorine contamination enriches virus-encoded metabolism and pesticide degradation associated auxiliary genes in soil microbiomes. ISME J 16:1397–1408. 10.1038/s41396-022-01188-w 35039616 10.1038/s41396-022-01188-w PMC9038774

[CR79] Zhu X, Tang L, Wang Z et al (2024) A comparative analysis of phage classification methods in light of the recent ICTV taxonomic revisions. Virology 594:110016. 10.1016/j.virol.2024.110016 38461619 10.1016/j.virol.2024.110016

[CR80] Zimmerman AE, Howard-Varona C, Needham DM et al (2020) Metabolic and biogeochemical consequences of viral infection in aquatic ecosystems. Nat Rev Microbiol 18:21–34. 10.1038/s41579-019-0270-x 31690825 10.1038/s41579-019-0270-x

